# Direct evidence of proximal tubular proliferation in early diabetic nephropathy

**DOI:** 10.1038/s41598-022-04880-1

**Published:** 2022-01-17

**Authors:** Noriko Uehara-Watanabe, Natsuko Okuno-Ozeki, Atsushi Minamida, Itaru Nakamura, Tomohiro Nakata, Kunihiro Nakai, Aya Yagi-Tomita, Tomoharu Ida, Kisho Ikeda, Takashi Kitani, Noriyuki Yamashita, Michitsugu Kamezaki, Yuhei Kirita, Satoaki Matoba, Keiichi Tamagaki, Tetsuro Kusaba

**Affiliations:** 1 Department of Nephrology, Graduate School of Medical Science, Kyoto Prefectural University of Medicine, 465 Kajii-cho, Kamigyo-ku, Kyoto, 602-8566 Japan; 2 Department of Cardiovascular Medicine, Graduate School of Medical Science, Kyoto Prefectural University of Medicine, Kyoto, Japan

**Keywords:** Kidney diseases, Diabetes complications

## Abstract

Kidney hypertrophy is a common clinical feature in patients with diabetes and is associated with poor renal outcomes. Initial cell proliferation followed by cellular hypertrophy are considered the responsible mechanisms for diabetic kidney hypertrophy. However, whether similar responses against hyperglycemia continue in the chronic phase in diabetes is unclear. We performed lineage tracing analysis of proximal tubular epithelia using novel type 2 diabetic mice with a tamoxifen-inducible proximal tubule-specific fluorescent reporter. Clonal analysis of proximal tubular epithelia demonstrated that the labeled epithelia proliferated in type 2 diabetic mice. Based on the histological analysis and protein/DNA ratio of sorted labeled tubular epithelia, there was no evidence of cellular hypertrophy in type 2 diabetic mice. Lineage tracing and histological analyses of streptozocin-induced type 1 diabetes also revealed that cellular proliferation occurs in the chronic phase of type 1 diabetes induction. According to our study, epithelial proliferation accompanied by SGLT2 upregulation, rather than cellular hypertrophy, predominantly occurs in the hypertrophic kidney in both type 1 and type 2 diabetes. An increased number of SGLT2+ tubular epithelia may be an adaptive response against hyperglycemia, and linked to the hyper-reabsorption of sodium and glucose observed in type 2 diabetes patients.

## Introduction

Kidney hypertrophy is a common clinical feature in patients with diabetes. Kidney size has been linked to the poor outcomes of diabetic nephropathy in both type 1 and type 2 diabetes^1–3^. Renal hypertrophy primarily occurs in the cortex of a diabetic kidney^4^, and is thought to increase the expression of tubular transporter and subsequent hyper-reabsorption^5,6^.

The mechanisms of kidney growth have been investigated using an experimental streptozocin(STZ)-induced type 1 diabetes model^6^. The growth of proximal tubular epithelia in response to hyperglycemic stimulus is a multistep process, i.e. initial proliferation and subsequent cellular hypertrophy^5–8^. Many molecular mechanisms are involved in this process, including hyperglycemia-induced oxidative stress, increases in growth factors, such as insulin growth factor 1 (IGF-1) and epidermal growth factor (EGF), and activation of renin-angiotensin systems^4^. Subsequently, cell cycle arrest in the G1 phase occurs through p27Kip1 induction, leading to cellular hypertrophy^7^. In the experimental STZ-induced type 1 diabetes model, this process occurs within several days after the onset of hyperglycemia^7^. However, due to a lack of studies, whether these processes occur in type 2 diabetes is unclear.

Regarding the STZ-induced diabetes model, which has been mostly used in the previous experiments for kidney growth, the cytotoxic effects of STZ are one of the major concerns surrounding the assessment of tubular epithelial behavior in vivo^9^. In rodent experiments, STZ is usually used for generating type 1 diabetes rodent models through its cytotoxic effects on the pancreatic beta cells^10^. STZ also induces proximal tubular epithelial injury in vivo in a dose-dependent manner and reduces membrane transporter expression, which occurs independent of blood glucose values^9,11^. As cell cycle arrest is one of the major phenotypes of injured renal tubular epithelia^12^, it is possible that the cell cycle arrest in tubular epithelia after STZ administration is induced by its cytotoxic effects.

For evaluating or visualizing cell division, especially in acute kidney injury, immunostaining of markers or labeling of proliferating cells by thymidine analogs are common experimental methods. However, these strategies are not sufficient for clearly demonstrating cell division in cells with a slower turnover, which is a major problem for visualizing the effects of cell proliferation on kidney growth in type 2 diabetes. Instead, to elucidate the molecular response against hyperglycemic stimuli, in vitro experiments using tubular epithelial cell lines have been applied. However, the phenotypes of tubular epithelia, including cell polarity and expression of membrane transporters, which are essential for determining cellular responses against stimuli in vivo, are lost in culture conditions^13^. In addition, unlike physiological conditions in the normal kidney, cultured tubular epithelia actively proliferate, making it difficult to mimic the diabetic condition in vitro.

In order to overcome these limitations and to directly address the roles of cellular proliferation and hypertrophy in the diabetic kidney in vivo, we performed lineage tracing analysis of terminally differentiated proximal tubular epithelia. Genetic lineage tracing is a powerful experimental method to understand the cell fate under different disease conditions and enables tracking of the progeny of labeled cell populations^14,15^. We generated type 2 diabetic model mice with a tamoxifen-inducible tdTomato reporter in terminally differentiated proximal tubular epithelial cells, and analyzed the behavior of this population under hyperglycemic conditions. In addition, we examined the molecular changes in sorted tubular epithelia in diabetic mice by flow cytometry.

## Results

### Generation of type 2 diabetic mice with a proximal tubule reporter

**Figure 1:**
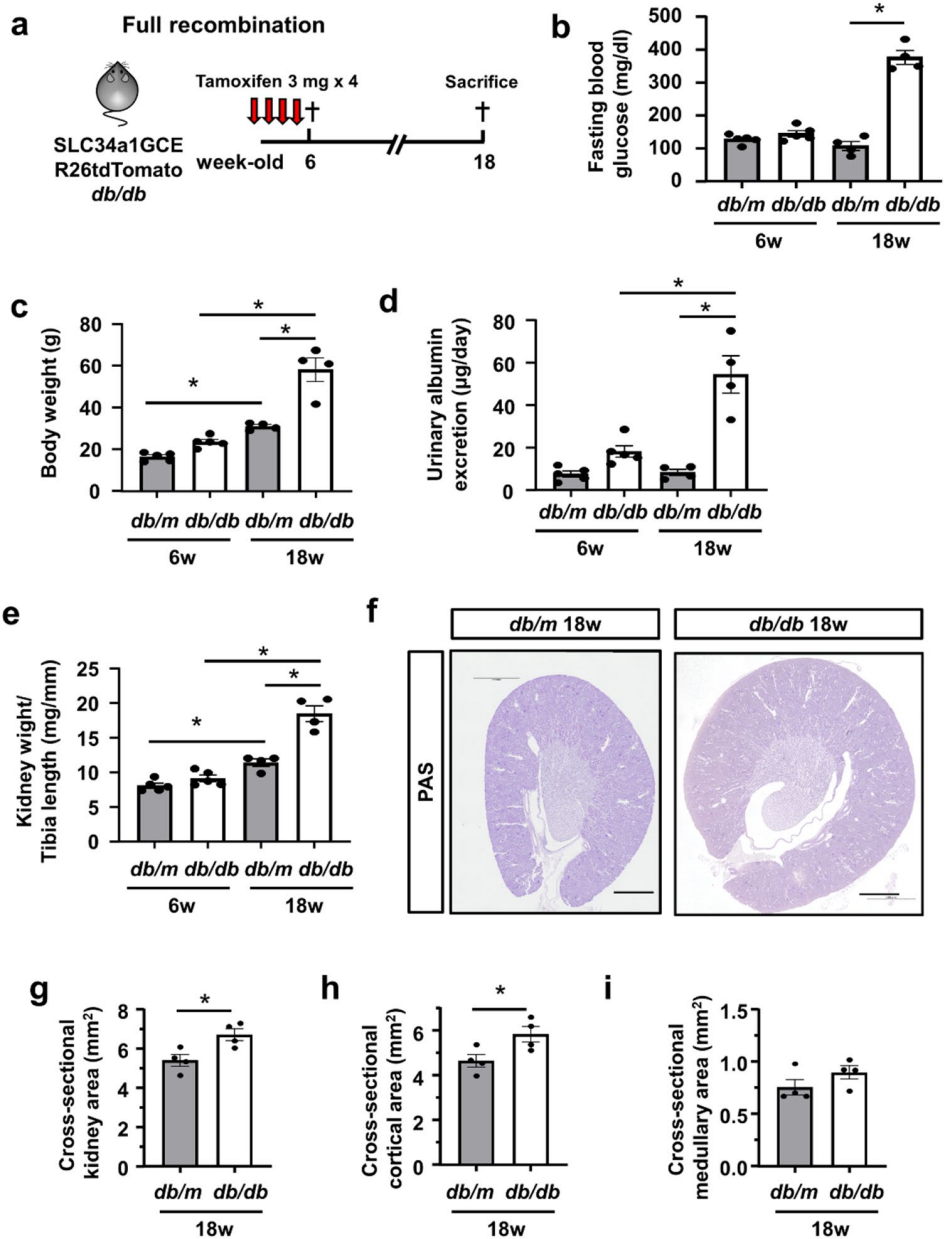
Kidney growth in *db/db* mice. (**a**) Experimental protocol. High-dose tamoxifen was injected 5 times into 6-week-old trigenic SLC34a1GFPCreERT2 (SLC34a1GCE), Rosa26-tdTomato (R26tdTomato), and *db/db* mice. Trigenic mice with SLC34a1GCE, R26tdTomato, and *db/m* were used as the non-diabetic control. (**b**,**c**) Fasting blood glucose and body weight were higher in *db/db* mice than in *db/m* mice at 18 weeks. (**d**) Urinary albumin excretion was higher in *db/db* mice than in *db/m* mice at 18 weeks. (**e**) Kidney weight/tibia length significantly increased in *db/db* mice at 18 weeks. (**f**) Representative PAS staining of kidney sections. (**g**–**i**) The cross-sectional kidney area (**g**) was larger in *db/db* mice at 18 weeks, especially in the cortex (**h**), but not in the medulla (**i**). N = 4–5 mice in each group. For all groups, data are means ± SEM, *p < 0.05, Bar = 1 mm in (**f**).

In order to investigate the proximal tubule-specific phenotypes in diabetic mice, we generated type 2 diabetic mice with a proximal tubule-specific reporter. We crossed *db/m* mice with mice carrying a proximal tubule-specific tamoxifen-inducible Cre (SLC34a1GCE) and the tdTomato reporter (R26tdTomato). In this transgenic mouse line, proximal tubules were exclusively labeled by tdTomato after tamoxifen injection^16^. After multiple injections of tamoxifen to label the proximal tubular epithelia at 6 weeks, the male mice were used for experiments (Fig. 1a). The body weights increased in both *db/m* and *db/db* mice during the observation period, but those in *db/db* mice significantly increased at 18 weeks (Fig. 1b). The blood glucose levels started to increase at 8 weeks in *db/db* mice, whereas those in *db/m* mice remained stable at around 100 mg/dl during the experimental period (Fig. 1c). Urinary albumin excretion was significantly higher in *db/db* mice (Fig. 1d). Kidney weight adjusted by tibia length was significantly higher in 18-week-old *db/db* mice (Fig. 1e).

Regarding kidney histology, the cross-sectional kidney area was significantly larger in *db/db* mice (Fig. 1f,g), which was due to expansion of the cortical area (Fig. 1h), not the medullary area (Fig. 1i). We also evaluated the glomerular area and found that glomeruli were much larger in *db/db* mice (Supplemental Fig. 1a,b), reflecting glomerular hyperfiltration.

**Figure 2:**
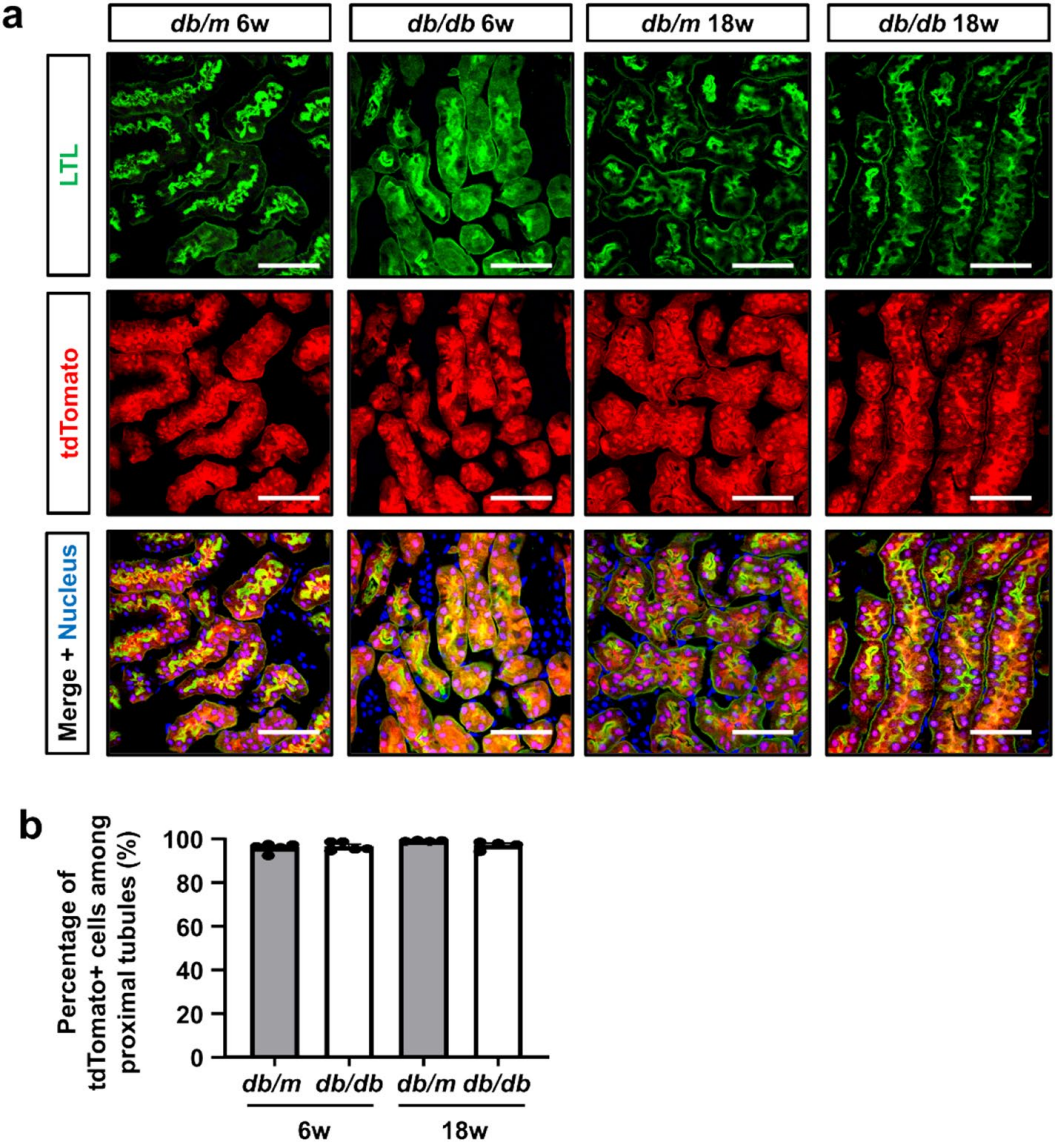
No participation of cells of extratubular origin in proximal tubules of *db/db* mice. (**a**) Representative images of fluorescence of LTL and tdTomato in kidney sections. Bar = 50 μm. (**b**) Quantification of the percentage of tdTomato+ cells among LTL + cells. There was no dilution of labeling of the tdTomato reporter in proximal tubules of *db/db* mice.

We next assessed the Cre-mediated recombination efficiency in newly generated diabetic mice with the proximal tubular epithelial reporter. In all mice groups, tdTomato+ cells were never found outside the proximal tubules. At 6 weeks of age, approximately 90% of cortical proximal tubular epithelial cells were labeled by tdTomato in both *db/m* and *db/db* mice (Fig. 2a). At 18 weeks of age, the percentages of labeled cells among proximal tubular epithelia were similar in both *db/m* and *db/db* mice, and there was no dilution of labeling frequency in 18-week-old *db/db* mice (Fig. 2b). Thus, cells from outside the tubules played no role in hypertrophy in the diabetic kidney.

### No evidence of epithelial cellular hypertrophy in *db/db* mice

**Figure 3:**
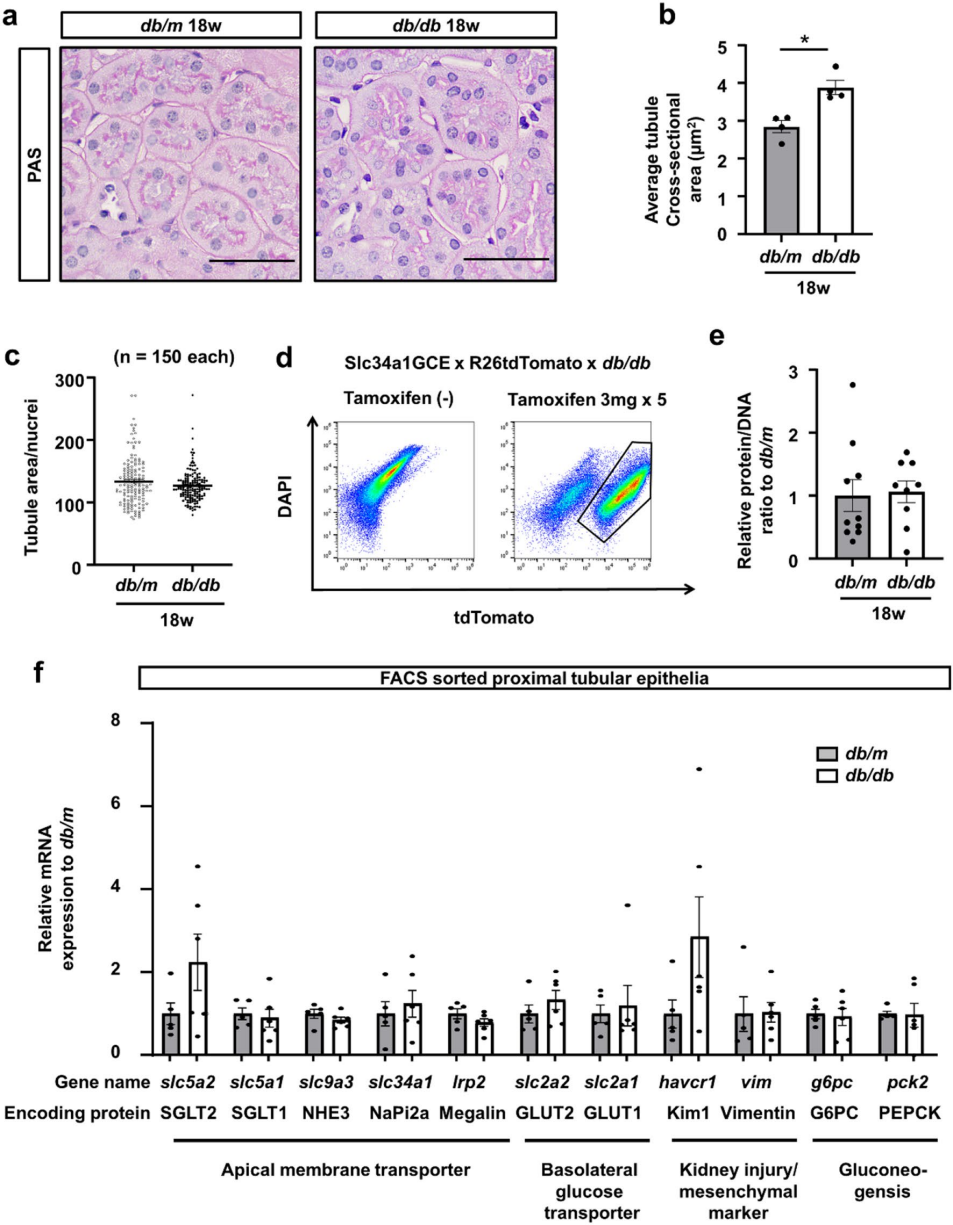
No evidence of cellular hypertrophy in proximal tubules of *db/db* mice. (**a**) Representative PAS staining of proximal tubules. (**b**) The average cross-sectional area of proximal tubules was larger in *db/db* mice at 18 weeks. (**c**) The ratio of tubular area to nuclei number in circular proximal tubules was calculated as a marker of single-cell size. There was no difference in cell size of proximal tubular epithelia between *db/db* and *db/m* mice. (**d**) Isolation of tdTomato+ tubular epithelial cells using FACS as described in the experimental scheme in Fig. 1a. (**e**) Protein DNA ratio of isolated tubular epithelia. There was no difference between *db/db* and *db/m* mice at 18 weeks. (**f**) qPCR of RNA from isolated tubular epithelia for the representative markers of apical membrane transporters (*slc5a2*, *slc5a1*,* slc9a3*, *slc34a1*, and *lrp2*), basolateral glucose transporters (*slc2a2* and *slc2a1*), tubular injury or mesenchymal markers (*havcr1* for tubular injury and *vim* for epithelial-mesenchymal transition), and gluconeogenesis (*g6pc* and *pck2*). For all groups, data are means ± SEM, Bar = 50 μm in (**a**).

We further calculated the average cross-sectional area of circular shaped proximal tubules (arbitrarily defined as minor/major axis > 0.8, Supplemental Fig. 2a,b) and found that the proximal tubules were enlarged in *db/db* mice (Fig. 3a,b). We measured the size of a single cell by calculating the ratio of the tubular area to the number of nuclei in circular proximal tubules on PAS staining, reflecting the occupied tubular area in each cell. The tubular area/nucleus ratio was not different between *db/m* and *db/db* mice (Fig. 3c). In order to further confirm that there was no cellular hypertrophy in proximal tubular epithelia in the diabetic kidney and to specifically analyze the phenotypes of proximal tubular epithelia, we collected the tdTomato+ tubular epithelia by fluorescence-activated cell sorting (FACS), and RNA and protein were extracted for further analysis (Fig. 3d). The protein/DNA ratio of isolated tdTomato+ epithelia, a marker of cellular hypertrophy, was not different between *db/m* and *db/db* (Fig. 3e). qPCR of isolated tubular epithelia demonstrated the RNA expression of *slc5a2*, encoding SGLT2, to be slightly higher in *db/db* mice, whereas that of proximal tubule-specific apical membrane transporters, such as *slc34a1* and *lrp2*, was not upregulated in *db/db* mice (Fig. 3f). Regarding *slc2a2* and *slc2a1*, basolateral glucose transporters, there were no differences between *db/m* and *db/db* mice (Fig. 3f). *Havcr1*, a kidney injury marker, was slightly upregulated in *db/db* mice; however, *vim*, a mesenchymal marker indicating the epithelial mesenchymal transition, was not upregulated in *db/db* mice (Fig. 3f). Western blot analysis using isolated tubular epithelia by FACS demonstrated the SGLT2 protein expression to be slightly higher in *db/db* mice (Supplementary Fig. S3a,b).

### Clonal analysis of proximal tubular epithelial cells in *db/db* mice

**Figure 4:**
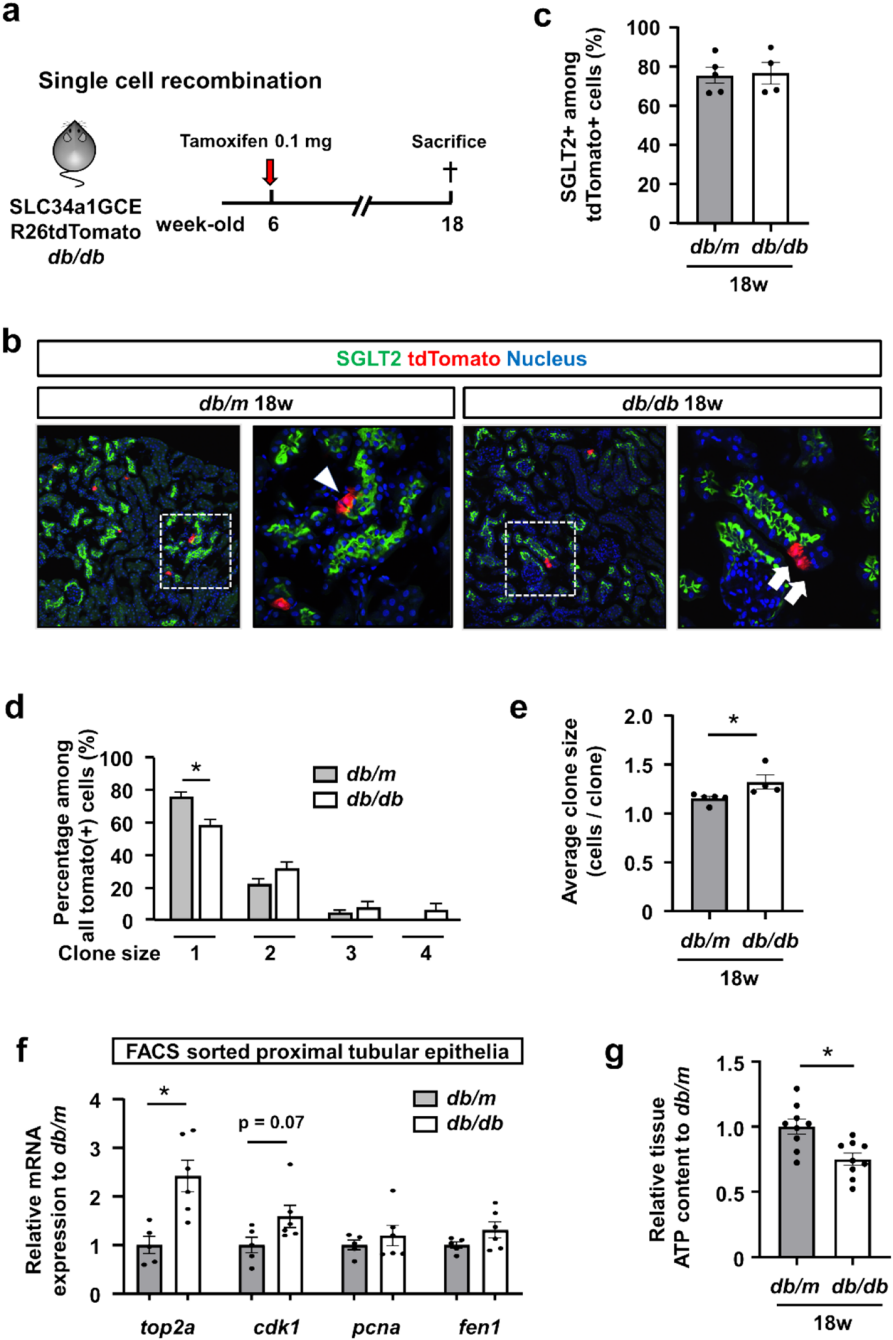
Clonal analysis of proximal tubular epithelial cells in *db/db* mice. (**a**) Experimental scheme. Single proximal tubular epithelial cells are labeled in SLC34a1GCE R26tdTomato *db/db* or *db/m* mice by low-dose tamoxifen injection at 6 weeks of age. (**b**) Representative images of immunostaining of SGLT2 (green) in *db/m* and *db/db* mice at 18 weeks. There was expansion of single tdTomato+ clones in *db/db* mice (arrows), whereas the majority of tdTomato+ cells remained as single-cell clones in *db/m* (arrowhead). (**c**) Rate of SGLT2 positivity among tdTomato+ epithelia. (**d**) Analysis of clone size and their frequency. Reduction of the frequency of single-cell clones and an increase in multicellular clones were observed in *db/db* mice. (**e**) The average clone size was larger in *db/db* mice. (**f**) qPCR of RNA from isolated tubular epithelia for the representative cell cycle markers. (**g**) Renal cortical tissue ATP level. For all groups, data are means ± SEM, *p < 0.05, Bar = 100 μm in (**B**).

In order to directly demonstrate epithelial proliferation in the diabetic kidney, we next performed in vivo clonal analysis of terminally differentiated proximal tubular epithelial cells. We performed sparse labeling by low-dose tamoxifen (0.05 mg) injection to generate single cell clones and sacrificed the male mice 12 weeks later (Fig. 4a). Approximately 80% of tdTomato+ cells were SGLT2+ in both *db/m* and *db/db* mice (Fig. 4b,c). tdTomato+ clones expanded in *db/db* mice, confirming active epithelial cell proliferation during the experimental period (Fig. 4b). The clone size was slightly larger in *db/db* mice (Fig. 4b,d). The average cell number per clone in each kidney was also greater in *db/db* mice (Fig. 4e). We further analyzed the clone size with/without SGLT2 expression separately. The clone size and average clone size of SGLT2+ epithelia were larger in *db/db* mice (Supplementary Fig. S4a,b); however, those of SGLT2- epithelia were not different between *db/m* and *db/db* mice (Supplementary Fig. S4c,d). To confirm the molecular response of cell proliferation in tubular epithelia, we evaluated the RNA expression of cell cycle regulatory molecules associated with the G1/S phase, including *pcna* and *fen1*, and the G2/M phase, including *cdk1* and *top2a*^17,18^, using tdTomato+ epithelia isolated by FACS, as shown in Fig. 2. The expression of these cell cycle related molecules was slightly higher in *db/db* mice, especially* top2a* (Fig. 4f). We next examined the tissue ATP content as a consequence of hyper-reabsorption of glucose and sodium through the increase in the number of SGLT2-expressing cells under diabetic conditions, and found that ATP content significantly decreased in *db/db* mice (Fig. 4g). Overall, our lineage tracing analyses demonstrated that cellular proliferation, not cellular hypertrophy, is responsible for the development of diabetic kidney hypertrophy in the chronic phase.

### Kidney growth in STZ-induced type 1 diabetic mice

**Figure 5:**
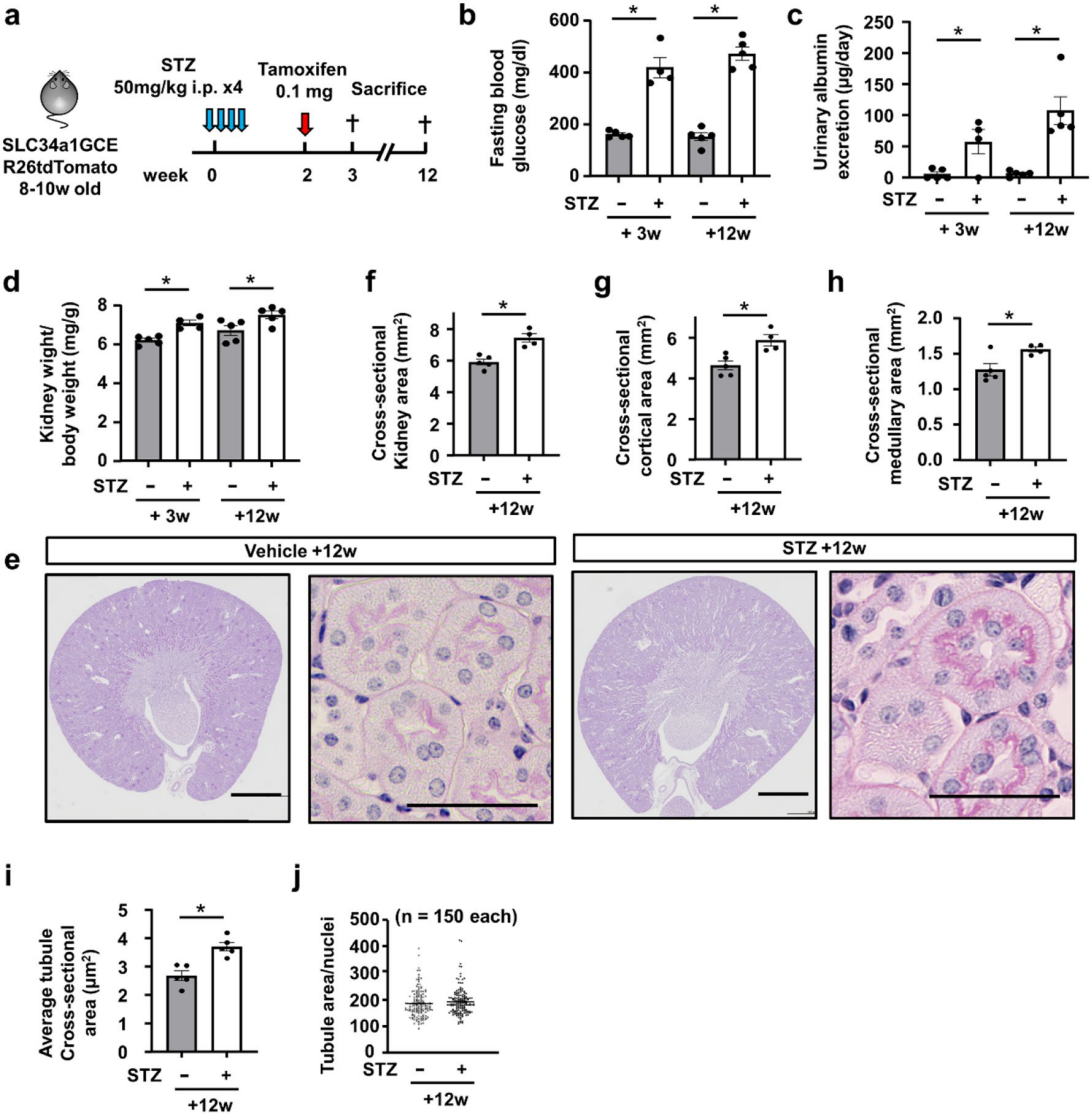
Kidney growth in STZ-induced type 1 diabetic mice. (**a**) Experimental scheme. Single proximal tubular epithelial cells were labeled by low-dose tamoxifen injection 2 weeks after 4 injections of 50 mg/kg of STZ. (**b**,**c**) Blood glucose and urinary albumin excretion were higher in STZ mice. (**d**) The kidney weight/body weight ratio significantly increased in STZ mice. (**e**) Representative PAS staining of kidney sections. (**f**–**h**) Cross-sectional kidney area. The cortical and medullary areas were larger in STZ mice. (**i**) The average cross-sectional area of proximal tubules was larger in STZ mice. (**j**) There was no difference in cell size of proximal tubular epithelia between STZ- and vehicle-treated mice. For all groups, data are means ± SEM, *p < 0.05, Bar = 1 mm in low power fields and 50 μm in high power fields of (**e**).

Based on the previous reports of a poor renal prognosis in type 1 diabetic patients with renal hypertrophy^1^, we examined whether tubular epithelial proliferation also led to kidney enlargement in the STZ-induced type 1 diabetic model mice. We injected low-dose STZ (40 mg/kg) or vehicle into bigenic male mice carrying SLC34a1GCE and R26tdTomato 5 times. After recovery from STZ-induced acute kidney injury, we performed sparse labeling by low-dose tamoxifen injection to generate single-cell clones 2 weeks after the last STZ injection and sacrificed the mice 12 weeks later (Fig. 5a). Fasting blood glucose significantly increased in STZ-treated mice (Fig. 5b). The amount of urinary albumin excretion and kidney weight adjusted by body weight were significantly higher in STZ-treated mice (Fig. 5c,d). Regarding kidney histology, the cross-sectional kidney area was significantly larger in STZ-treated mice (Fig. 5e,f), which was due to expansion of the cortical area (Fig. 5g) and medullary area (Fig. 5h). We further calculated the average cross-sectional area of proximal tubules and found that the proximal tubules increased in size in STZ-treated mice (Fig. 5e, 5i). The tubular area/nucleus ratio was not different between the groups (Fig. 5j). We also evaluated the glomerular area and found that glomeruli were larger in STZ-treated mice (Supplemental Fig. S5a,b).

### Clonal analysis of proximal tubular epithelial cells in STZ-induced type 1 diabetic mice

**Figure 6:**
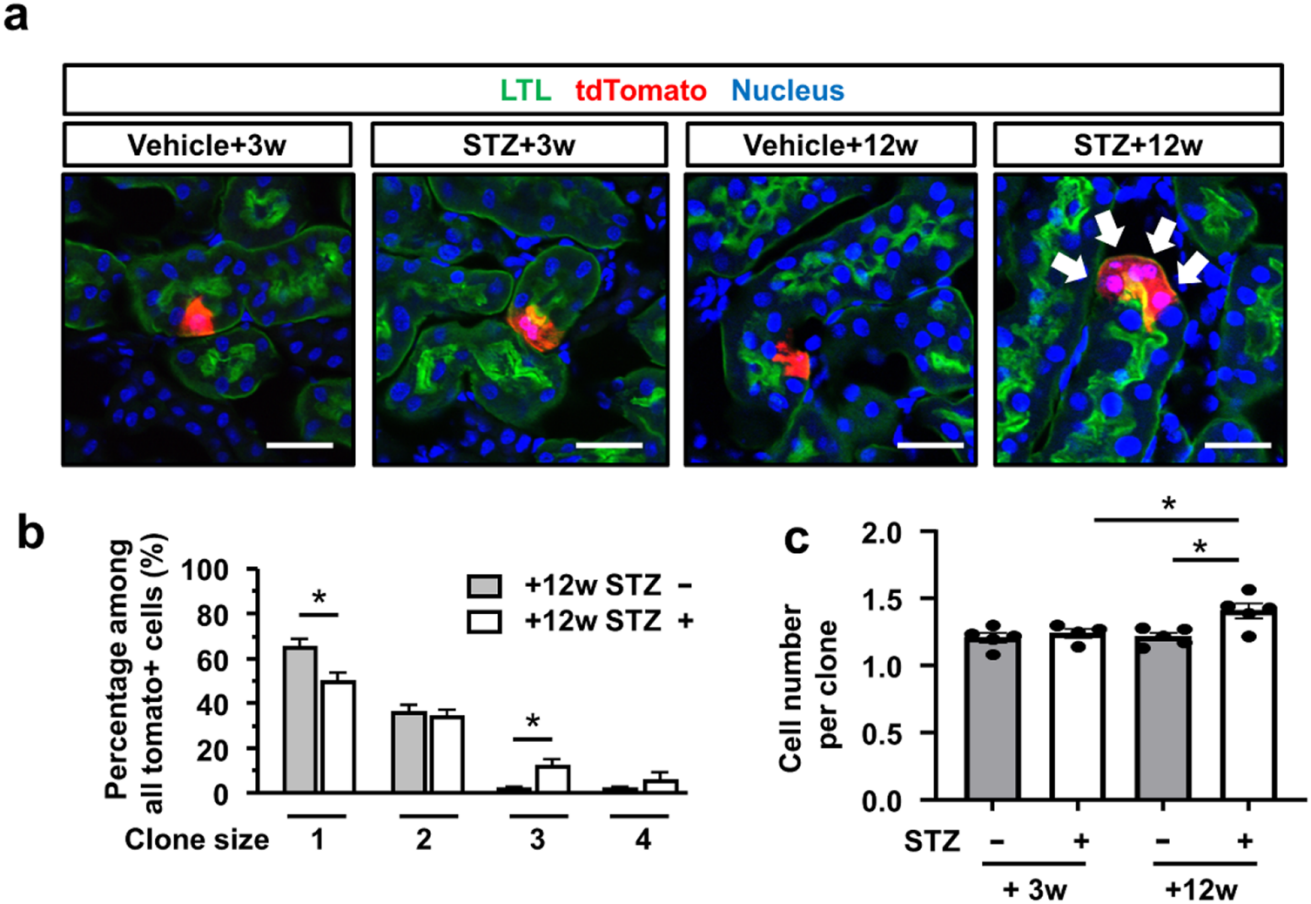
Clonal analysis of proximal tubular epithelial cells in STZ-induced type 1 diabetic mice. (**a**) Representative images of immunostaining of LTL (green) in STZ- or vehicle-injected mouse kidneys 3 or 12 weeks after STZ injection. There was expansion of tdTomato+ clones in STZ mice (arrowheads). (**b**) Analysis of clone size and their frequency. Reduction of the frequency of single cell clones and an increase in multicellular clones were observed in STZ injected mice. (**c**) The average clone size was larger 12 weeks after STZ injection. N = 4 to 5 mice per group. For all groups, data are means ± SEM, *p < 0.05, Bar = 100 μm in (**a**).

In vivo clonal analysis of terminally differentiated proximal tubular epithelial cells revealed that tdTomato+ clones expanded in STZ-treated mice, reflecting active epithelial cell proliferation during the experimental period (Fig. 6a). In STZ-treated mice, the percentage of single-cell clones was lower and that of multiple-cell clones was larger than in vehicle-treated mice (Fig. 6b). The average cell number per clone in each kidney was also larger in STZ-treated mice than in vehicle-treated mice, whereas there was no difference in the initial phase (Fig. 6c). Thus, cell proliferation rather than cellular hypertrophy plays a major role in kidney enlargement in type 1 diabetes.

## Discussion

This study addressed the cellular responses of proximal tubular epithelial cells in the early diabetic kidney using lineage tracing analysis of terminally differentiated proximal tubular epithelia. Our clonal analysis of proximal tubular epithelia labeled at the single-cell level demonstrated active cell proliferation in the kidney in both type 1 and type 2 diabetic mouse models. Analysis using FACS-sorted labeled proximal tubular epithelia revealed no evidence of cellular hypertrophy in proximal tubular epithelia in type 2 diabetes. Therefore, proximal tubular epithelial proliferation, rather than cellular hypertrophy, occurs during kidney growth under diabetic conditions. As such, cellular proliferation accompanying an increase in SGLT2 expression is an adaptive response against the increase in filtered glucose through the glomerulus, thereby increasing the reabsorption of urinary glucose.

The responsible cellular mechanism leading to kidney growth was heavily debated in the early 2000’s. In vivo experiments using thymidine analogs and in vitro experiments revealed that the cellular response after hyperglycemic stimuli-induced cellular hypertrophy plays a major role in kidney growth^7,19^. Due to a lack of appropriate methods for tracking the fates of proximal tubular epithelia long-term in diabetic mice, there have been no updates in this field for almost 20 years. However, the direct evidence of the proliferation of proximal tubular epithelial cells in the hypertrophic diabetic kidney provided in this study is not consistent with previous reports arguing that cellular hypertrophy is a major player in this phenomenon. This discrepancy may be attributed to the following.

First, the previous experiments focusing on the kidney growth in diabetes were mainly performed using STZ-induced type 1 diabetes model mice because of the convenience of identifying the onset of diabetes^7,19–22^. STZ is a member of a group of alkylating antineoplastic drugs and is widely used for the treatment of neuroendocrine tumors due to its cytotoxic effects^23^. STZ is taken up by pancreatic beta-cells through GLUT2, resulting apoptosis and type 1 diabetes^10,24^. However, as GLUT2 is abundantly expressed in the basolateral membrane of proximal tubular epithelia, STZ can induce tubular epithelial injury, which is observed even though hyperglycemia is not evident in STZ-injected mice^9,11,25^. In the case of DNA damage in tubular epithelia, cell cycle arrest is introduced to prevent further DNA damage, resulting in a higher protein/DNA ratio. As it is difficult to distinguish whether cell cycle arrest was induced by hyperglycemic stimuli or by STZ-induced DNA damage^25^, careful interpretation of cell cycle arrest and subsequent cellular hypertrophy after STZ injection is required.

Second, turnover of tubular epithelia in the kidney is markedly slow and only 0.5 to 1.0% of tubular epithelia were positive for Ki-67, a marker of cell division, under normal conditions^16^. As diabetes is a chronic disease condition and a longer period is required for notable phenotypes, it is difficult to visualize differences in positivity of cell proliferation markers in mice with or without diabetes. The observation period in most previous reports using STZ-induced type 1 diabetic mice was within 2 weeks, which is not sufficient to reach the conclusion that cellular proliferation did not contribute to kidney growth, especially in type 2 diabetes. Our lineage tracing analysis of genetically labeled proximal tubular epithelia and fate tracking for a longer period can overcome this limitation.

We analyzed the gene expression of several transmembrane transporters using FACS-sorted proximal tubular epithelia, and found that SGLT2 expression was slightly upregulated, whereas basolateral glucose transporters, GLUT1 and GLUT2, were not. This is inconsistent with previous studies reporting the upregulation of both SGLT2 and GLUT2 expression in the primary culture of urine-derived proximal tubular epithelia cells from diabetic patients^26^. As proximal tubular epithelia are highly polarized cells under normal conditions in vivo, the expression patterns and functions of membrane transporters are different between in vitro cultured conditions and in vivo physiological conditions^13,27^, which may be problematic for the analysis of diabetic conditions in vitro*.* For example, SGLT2 expression is observed at the apical membrane of tubular epithelia in vivo*,* but glucose transport is reduced in in vitro cultured conditions even if SGLT2 mRNA is overexpressed in transfected oocytes^28^. In addition, analysis using a two-chamber culture system clarified that only high basolateral glucose exposure increased SGLT2 expression in a proximal tubular epithelia cell line^13^. Thus, cellular polarity is essential to maintain the expression of membrane transporters, including SGLT2, on the cellular apical surface, and careful interpretation is required for analysis in vitro. Our analysis using freshly isolated proximal tubules by FACS reflects the gene expression in vivo and demonstrated the upregulation of SGLT2 expression in individual proximal tubular epithelial cells, albeit lower than that in previous reports^26^.

In terms of active tubular proliferation and increased expression of SGLT2 in each proximal tubular epithelial cell, these are factors underlying the increase in SGLT2 expression in kidney tissue, which may cause the higher reabsorption of urinary glucose. Due to its anatomical structure and physiological function of high sodium reabsorption, kidneys are borderline hypoxic at baseline and are therefore likely to respond to further decreases in oxygen availability such as by releasing erythropoietin^29^. In response to hyperglycemic stimuli, increased reabsorption of glucose and sodium through SGLT2 leads to a higher oxygen demand due to overactivation of basolateral Na–K ATPase^30^. In previous reports, tissue hypoxia at the renal cortex was noted in mouse models of both type 1 and type 2 diabetes, which was ameliorated by pharmacological SGLT2 inhibition^31^. Considering the hyper-reabsorption of sodium and glucose caused by the overexpression of SGLT2 in kidney tissue, kidney growth may be related to higher oxygen consumption and subsequent tissue hypoxia, which synergistically cause the acceleration of tissue damage in the diabetic kidney, resulting in its poor renal prognosis in diabetic patients.

In conclusion, we clarified the importance of cellular proliferation accompanying SGLT2 upregulation in the development of early diabetic nephropathy. Control of blood glucose and blood pressure is essential for preventing diabetic nephropathy, and SGLT2 inhibitors markedly reduce the risk of developing renal events. Although we demonstrated that the proliferation of SGLT2+ proximal tubular epithelial cells plays a major role in kidney growth, the precise molecular mechanisms remain unknown, which is a potential limitation of our study. Considering the poor prognosis of enlarged kidneys in diabetic patients, the molecular mechanisms of this phenomenon may be an attractive therapeutic target for preventing diabetic nephropathy, but further studies are required.

## Methods

### Animal experiments

We recently generated mice (SLC34a1GCE) with the CreERT2 cassette in the SLC34a1 locus, which enables expression of the Cre recombinase in the proximal tubules after tamoxifen injection^16^. SLC34a1GCE mice were crossed with the R26tdTomato reporter mice, in which tdTomato is expressed after Cre-mediated recombination of the floxed stop cassette to obtain bigenic offspring. We further crossed these bigenic mice with the non-diabetic C57BLKS/J Iar-m+/+Lepr^db^ (*db/m*) mice purchased from Oriental Bio Service Inc. (Kyoto, Japan), and obtained diabetic C57BLKS/J Iar—+Lepr^db^/+Lepr^db^ (*db/db*) mice carrying SLC34a1GCE and the R26tdTomato reporter (SLC34a1GCE/R26tdTomato/*db/db*). Mice were housed under a 12-h light/dark cycle with free access to tap water and standard chow (CE-2, CLEA Japan, Inc., Tokyo, Japan). For genetic labeling, tamoxifen (Sigma-Aldrich Co., LCC., St. Louis, MO) was dissolved in 3% (vol/vol) ethanol containing corn oil (Sigma Aldrich) at a concentration of 10 mg/ml. Tamoxifen was injected intraperitoneally at the indicated dose either once for single cell recombination or 5 times for full recombination. At the age of 18 weeks, male mice were anesthetized with isoflurane and euthanized. After weighing the kidney, the kidneys were cut into samples for further analysis.

For lineage tracing analysis of proximal tubular epithelial cells in the STZ-induced type 1 diabetic model mice, we injected low-dose STZ (40 mg/kg, Cayman Chemical, MI, USA) or vehicle into bigenic male mice with SLC34a1GCE and R26tdTomato 5 times. One week after the STZ injection, mice with blood glucose levels that increased to higher than 350 mg/dl were used in experiments. After waiting for recovery from STZ-induced acute kidney injury, 0.05 mg of tamoxifen was injected intraperitoneally for single-cell recombination.

Each experimental group contained at least 5 male mice. All experiments were approved by the Experimental Animals Committee, Kyoto Prefectural University of Medicine, and were performed in accordance with the institutional guidelines and Guidelines for Proper Conduct of Animal Experiments by the Science Council of Japan and the ARRIVE guidelines.

### Metabolic data

Body weights and blood glucose levels were measured at 17:00 every 2 weeks. Blood glucose levels were measured by a glucometer (Glutest Every, Sanwa Kagaku Kenkyusho Co., Ltd., Aichi, Japan).

Regarding 24-h urine collection, mice were placed individually into metabolic cages (KN-645, Natsume Seisakusho Co., Ltd., Tokyo, Japan) every 4 weeks. Urine albumin and creatinine levels were measured using an immunoturbidimetric method (Oriental Yeast Co., Ltd., Tokyo, Japan) and enzyme-linked immunosorbent assay (Nikken Seil Co., Ltd., Shizuoka, Japan), respectively.

### Tissue preparation and histological analysis

For frozen sections, kidneys were fixed with 4% paraformaldehyde in PBS for 1 h on ice, incubated in 30% (vol/vol) sucrose at 4 °C overnight, and embedded in Optimum Cutting Temperature Compound (Sakura FineTek, Tokyo, Japan). Then, 7-μm sections were cut. For paraffin sections, the kidneys were fixed with 4.0% paraformaldehyde and embedded in paraffin. Paraffin-embedded tissues were cut into 4-μm-thick sections. Morphological evaluations of the kidneys were performed by periodic acid-Schiff (PAS) staining under standard conditions. For the evaluation of kidney hypertrophy, the cortical area of the short axis section, which was cut at the middle of the kidney, was measured. For the quantification of glomerular size, the glomerular area in the same section, which contains approximately 100 glomeruli, was measured and the average glomerular size was calculated.

For the quantification of proximal tubular hypertrophy, the total area of cortical proximal tubules (the remaining cortical area after subtracting the area of glomeruli, of tubules without PAS+ brush border and of large vessels) was measured. Thereafter, the average cross-sectional area of each proximal tubule was calculated using the following formula: Cross-sectional area of proximal tubule = Total area of cortical proximal tubules / the total number of cross-sections of proximal tubules (approximately 1500 tubules per mouse).

For the quantification of cellular hypertrophy, we measured the area of circular sections of 50 randomly selected proximal tubules in each mouse in a blinded manner. Circular sections of proximal tubules were arbitrarily defined as minor/major axis > 0.8 (Supplemental Fig. S2a). The size of a single proximal tubular epithelial cell was calculated as the ratio of these areas to the number of nuclei (Supplemental Fig. S2b).

Histological quantification was performed using a BZ-X700/BZ-X710 microscope (Keyence Corporation, Osaka, Japan) and ImageJ software (National Institutes of Health, Bethesda, MD).

### Immunofluorescence analysis

For immunofluorescence, frozen sections were rehydrated and permeabilized with 0.5% Triton X-100 in PBS for 5 min. Samples were blocked with 10% normal goat serum in PBS and sequentially incubated with the primary antibodies shown in Supplemental Table S1 for 1 h, followed by incubation with Alexa Fluor 488-conjugated secondary antibodies (Supplemental Table S1) for 1 h. Nuclear counterstaining was performed using DAPI or DRAQ5 (DR50050; BioStatus, Leicestershire, UK; 1:2000), followed by mounting in Prolong-Gold (Thermo Fisher Scientific). Images were obtained by confocal microscopy (FV1000; Olympus, Tokyo, Japan).

Lineage tracing analyses of terminally differentiated proximal tubular epithelial cells were performed as described previously^16^. For dilution analysis, tdTomato+ cells among LTL + cells were quantified from 5 of 25 consecutive non-overlapping cortical fields in each kidney under high magnification (n = 5). These 5 fields were randomly selected in a blinded manner. For clonal analysis, the number of consecutive tdTomato+ cells in more than 10 HPF images of each kidney sections in randomly selected cortical fields was counted (n = 5).

### Separation of tdTomato-positive proximal tubular epithelia using FACS and protein / DNA ratio measurement.

The kidney cortex was minced and a single-cell suspension was generated using research grade Liberase TL (5401020001: Sigma-Aldrich) and 60 units/ml of DNAse Inhibitor (D5025: Sigma-Aldrich) for 30 min at 37 °C. Cells were washed twice with MACS® Separation Buffer (Miltenyi Biotec GmbH Bergisch Gladbach, Germany), filtered through 70- and 40-μm cell strainers, resuspended in autoMACS® Running buffer (Miltenyi Biotec GmbH) with 1000:1 DAPI (1 mg/mL), and subjected to FACS using SH800 (SONY, Tokyo, Japan). Dead cells (DAPI+) were excluded during FACS. TdTomato+ DAPI- cells were collected in DMEM and 10% FBS. RNA, DNA, and protein were extracted using NucleoSpin® TriPrep (TAKARA Bio Inc, Shiga Japan). DNA concentrations in the extract were measured using NanoDrop 2000 (Thermo Fisher Scientific, Waltham, MA, USA). The protein concentration in the extract was measured using the Protein Quantification Assay (MACHEREY–NAGEL, Germany). The protein-to-DNA ratio was calculated and data are presented as percentage increases relative to *db/m*.

### RNA extraction and real-time quantitative PCR.

Total RNA extraction and real-time quantitative PCR were performed as described previously^17^. Total RNA was isolated from the kidneys or FACS-sorted cells using TRIzol (Life Technologies, Inc., Carlsbad, CA) and Direct-zolTM RNA MiniPrep (Zymo Research Corporation., Irvine, CA) according to the manufacturer’s protocol. Subsequently, complementary DNA (cDNA) was generated using a Prime Script reverse transcription (RT) reagent kit (RR0471A Takara Bio Inc., Shiga, Japan), and real-time PCR was performed using KAPA SYBR Fast universal (KK4602: Kapa Biosystems, Wilmington, MA) and a Thermal Cycler Dice Real Time System (Takara Bio Inc., Shiga, Japan). All reactions were performed in duplicate. An initial denaturation step was performed for 10 min at 95 °C, which was followed by 45 cycles of amplification at 95 °C for 10 s, 62 °C for 10 s, and 72°C for 30 s. Gene expression was quantified using *gapdh* as an internal control. The primers used are listed in Supplemental Table 2.

### Western blot analysis

Western blot analysis of FACS-sorted cells was performed as described previously^17^. After extraction of total cell lysates by NucleoSpin® TriPrep, proteins were denatured by heating for 5 min at 95 °C and separated by SDS-PAGE. Subsequently, proteins were transferred onto polyvinylidene difluoride (PVDF) membranes (Immobilon-P IPVH00010: Millipore, MA, USA). After blocking in 5% non-fat milk in TBS/0.1% Tween20 for 1 h at room temperature, the membrane was incubated with the corresponding primary antibody for SGLT2 (ab85626, abcam) overnight at 4 °C. After washing with TBS/0.1% Tween20, secondary peroxidase-conjugated anti-mouse or anti-rabbit antibodies were added (7074S; Cell Signaling Technology, Boston, MA; 1:3000 at room temperature). Chemiluminescence was detected using an ECL select Western blot detection reagent (RPN2235: GE Healthcare UK Ltd, Amersham Place, England) or Clarity Max western ECL substrate (1,705,062: Bio-Rad Laboratories, Inc. Hercules, CA, USA). Signal intensities were evaluated using ImageJ software (National Institutes of Health, Bethesda, MD).

### Measurement of ATP levels in renal tissues

ATP concentrations in renal cortex samples were measured using an ATP-bioluminescent assay with the ''Tissue'' ATP assay Kit (TOYO B-Net Co. Ltd, Tokyo, Japan) according to the manufacturer’s instructions. ATP concentrations are expressed as the ratio of ATP concentration to tissue volume.

### Statistics

Results are expressed as the mean ± SEM. Each experiment was performed using at least five mice per group. Statistical analysis was carried out using the unpaired t-test for comparison of two variables, and by analysis of variance and Dunnett’s post hoc test for comparison of multiple variables. P-values < 0.05 were considered significant.

## Supplementary Information

Supplementary Information.
