# Supplementary material for: Direct evidence of proximal tubular proliferation in early diabetic nephropathy
Source: Sci Rep. 2022 Jan 17;12:778. doi: 10.1038/s41598-022-04880-1 (PMC8763925; doi:10.1038/s41598-022-04880-1)
Supplement: Supplementary file 1 — Supplementary Information. [file 41598_2022_4880_MOESM1_ESM.docx]

**Supplementary Information**

**Direct evidence of proximal tubular proliferation in early diabetic nephropathy**

Noriko Uehara-Watanabe, Natsuko Okuno-Ozeki, Atsushi Minamida, Itaru Nakamura, Tomohiro Nakata, Kunihiro Nakai, Aya Yagi-Tomita, Tomoharu Ida, Kisho Ikeda, Takashi Kitani, Noriyuki Yamashita, Michitsugu Kamezaki, Yuhei Kirita, Satoaki Matoba, Keiichi Tamagaki, and Tetsuro Kusaba

**Supplemental Figure 1**. Histological analysis of glomerulus in *db/db* mice.


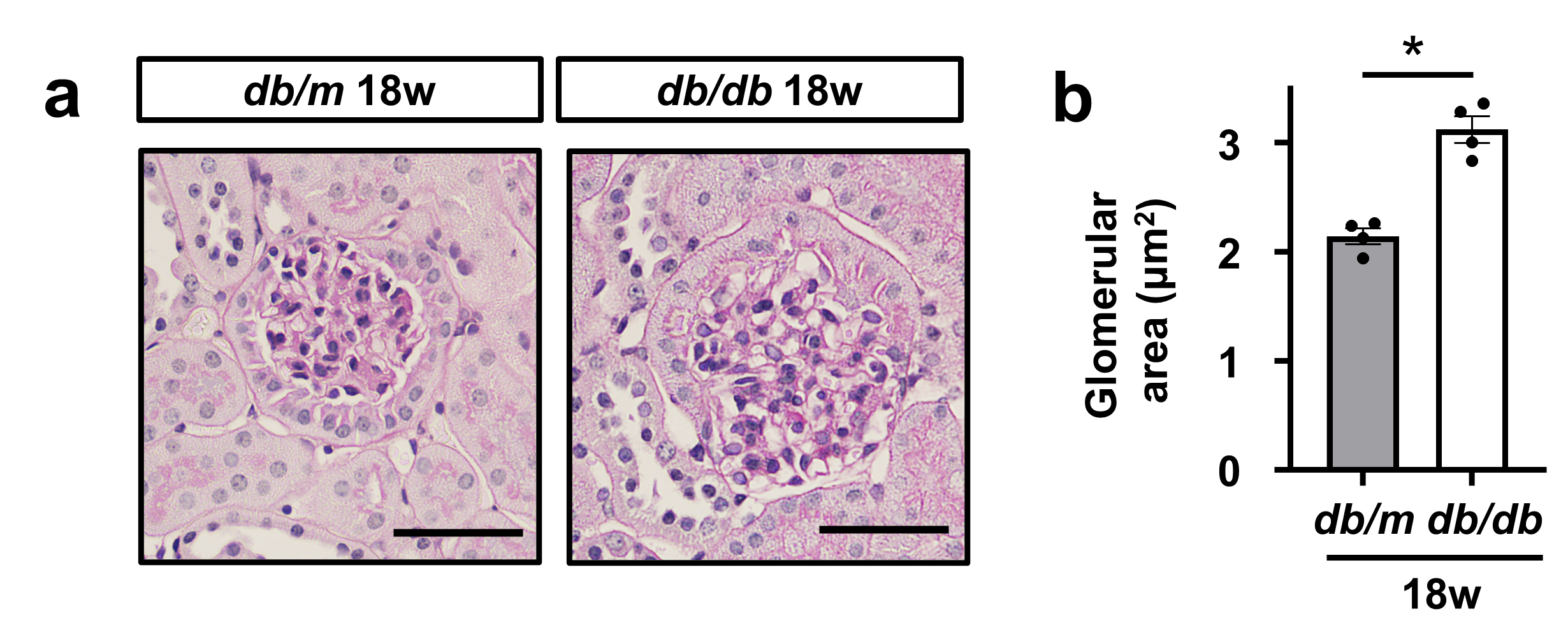
(a) Representative pictures of PAS staining of glomerulus. (b) Average glomerular area was larger in *db/db* mice at 18 weeks-old. N = 4-5 mice in each group. Data are means ± SEM, * p < 0.05, Bar = 50 μm in (A).

**Supplementary Figure 2**. Methods of histological analysis of tubular and cellular hypertrophy in proximal tubules.

(a) Arbitrarily definition of circular proximal tubules for quantification of tubular hypertrophy as major (length a) / minor (length b) axis > 0.8. (b) Quantification of the size of single proximal tubular epithelial cell was calculated as occupied cellular area (area c) / nuclei number.

**
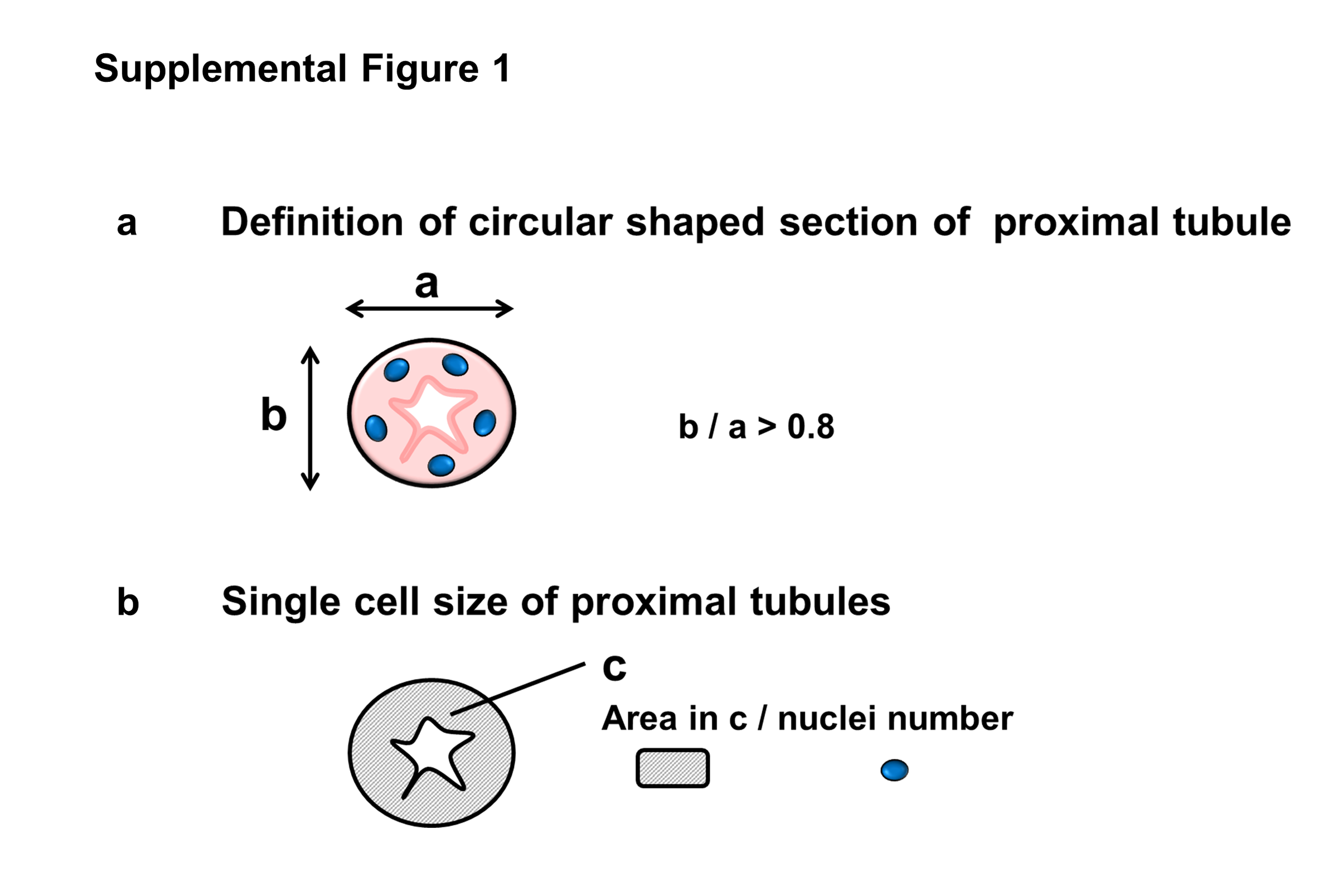
**

**Supplementary Figure 3**. Western blot analysis of SGLT2 in proximal tubular epithelial cells.

(a) Western blot of protein lysate from isolated tubular epithelia for SGLT2 and GAPDH. Representative pictures from n=3. (b) Optical density of SGLT2 bands were normalized against those of GAPDH. The normalized density of the samples from the control mice was arbitrarily set to 1. For both groups, data are means ± SEM.


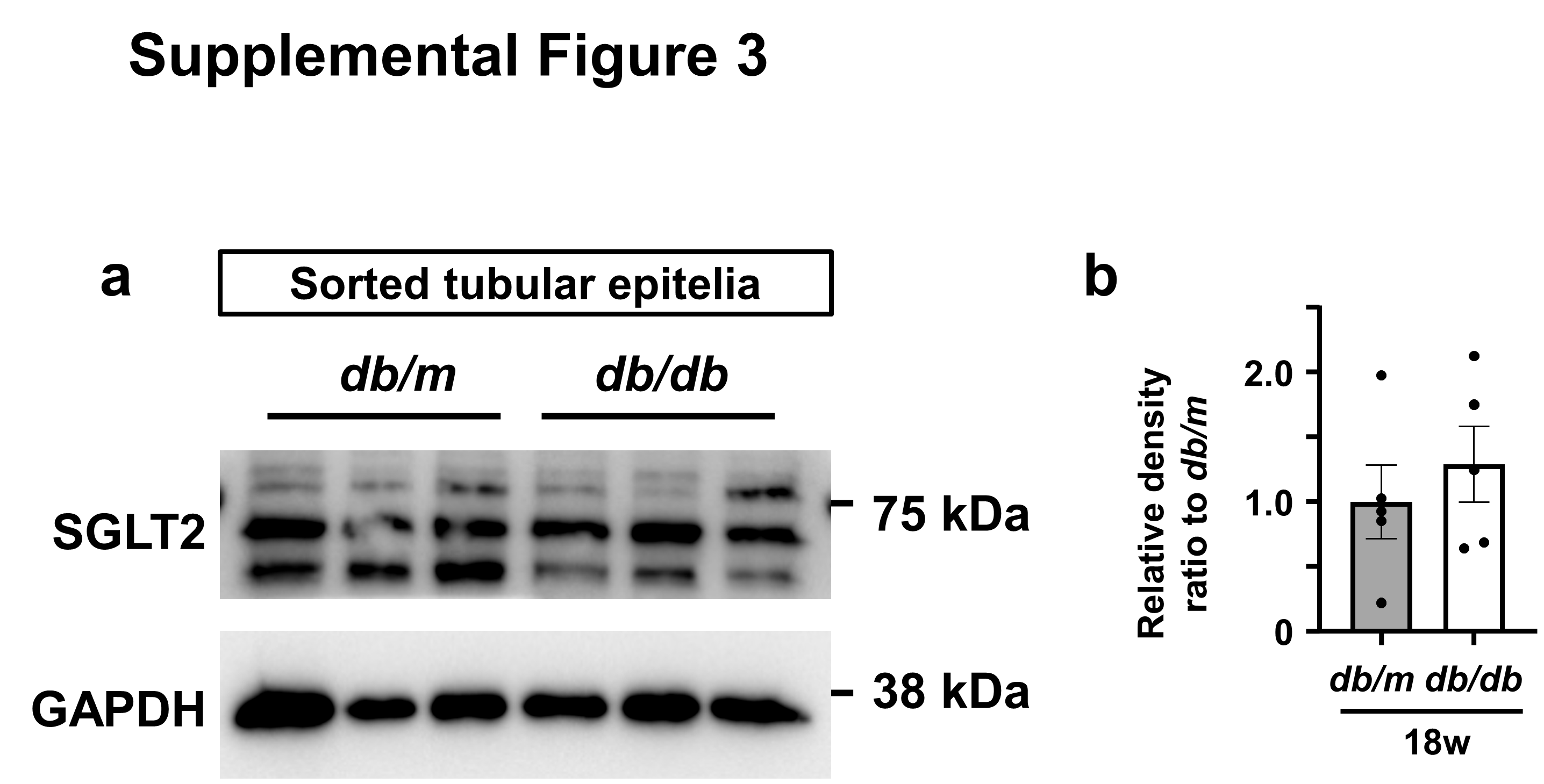


**Supplementary Figure 4**. Separate analyses of clone size and their frequency according to SGLT2 positivity in *db/db* mice.

(a, c) Reduction of the frequency of single-cell clones and an increase in multicellular clones were observed only for SGLT2+ cells of *db/db* mice. (b, d) The average clone size was larger for the SGLT2+ cells of *db/db* mice, whereas there was no difference in the average clone size between *db/m* and *db/db* mice. For all groups, data are means ± SEM, * p < 0.05.

**
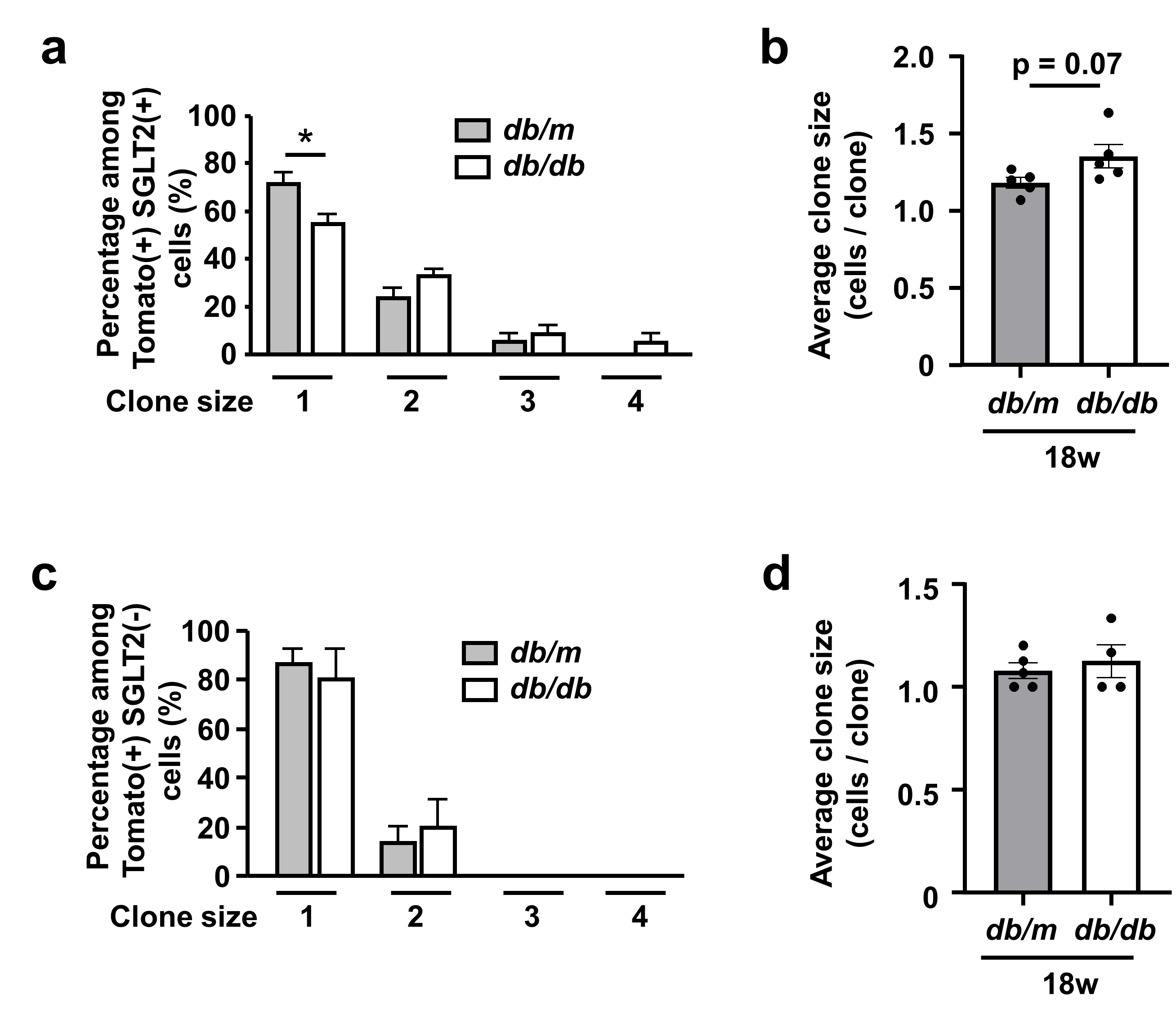
**

**Supplementary Figure 5**. Histological analysis of glomerulus in STZ-induced type 1 diabetic mice.

(a) Representative pictures of PAS staining of glomerulus. (b) Average glomerular area was larger in STZ-induced diabetic mice. N = 4-5 mice in each group. Data are means ± SEM, * p < 0.05, Bar = 50 μm in (A).


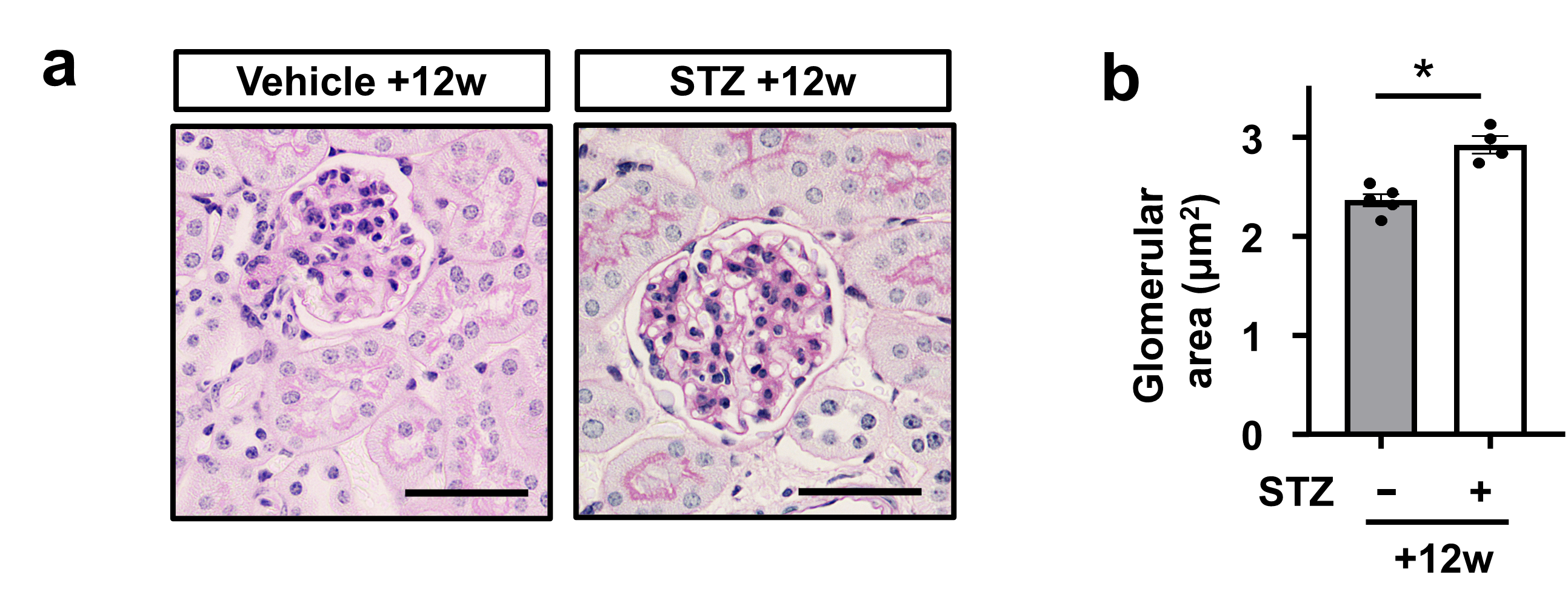


**Supplementary Figure 6**. Validation of SGLT2 antibody and original images of the blots in Supplementary Figure 3.

**
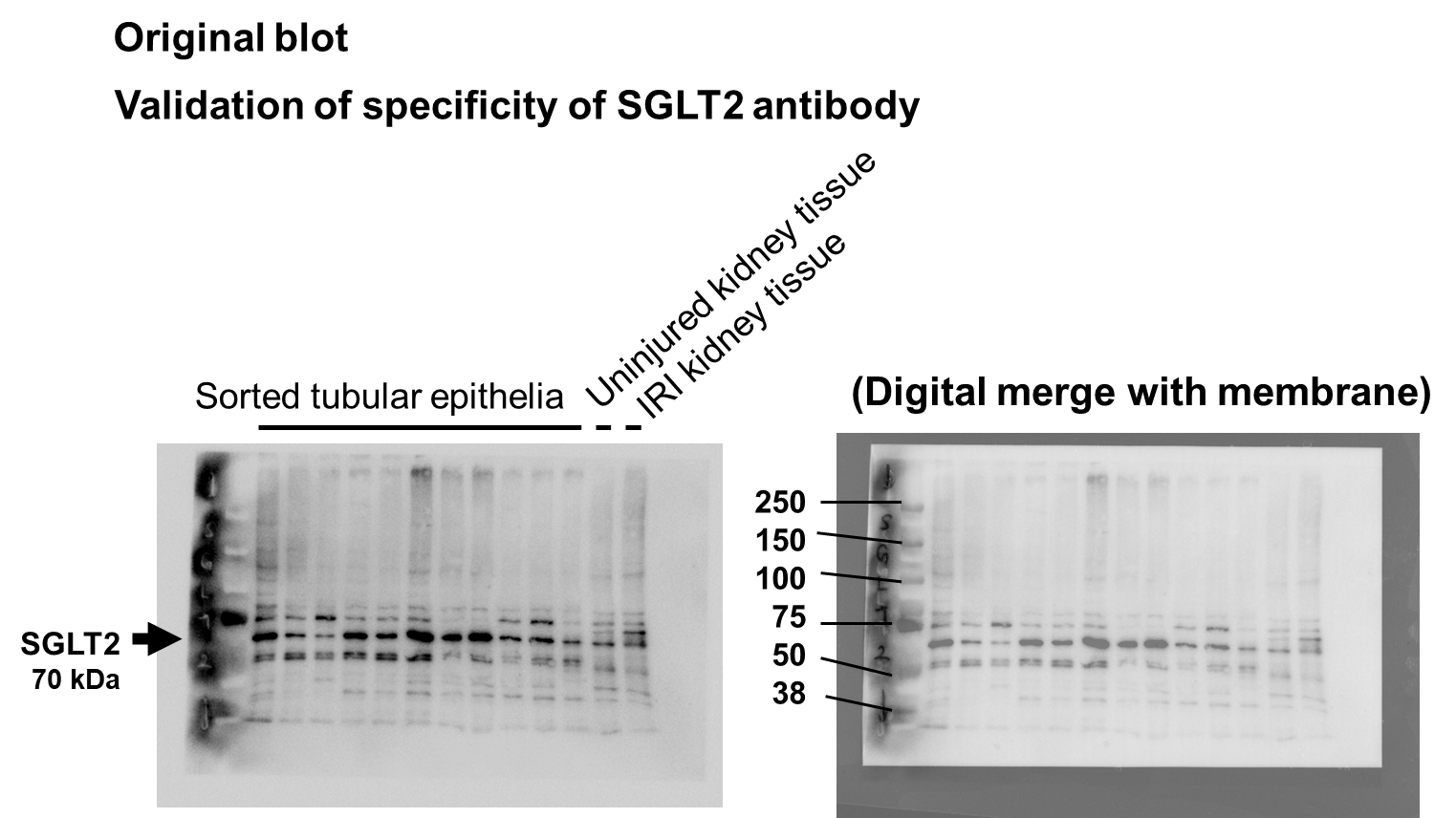
**After confirming that no extra bands were found below 50 kDa in the preliminary full-blotting picture for SGLT2 (upper panels), immunoblotting of SGLT2 (70 kDa) and GAPDH (37 kDa) was performed using the same PVDF membrane which was cut at 50 kDa marker (lower panels).

**
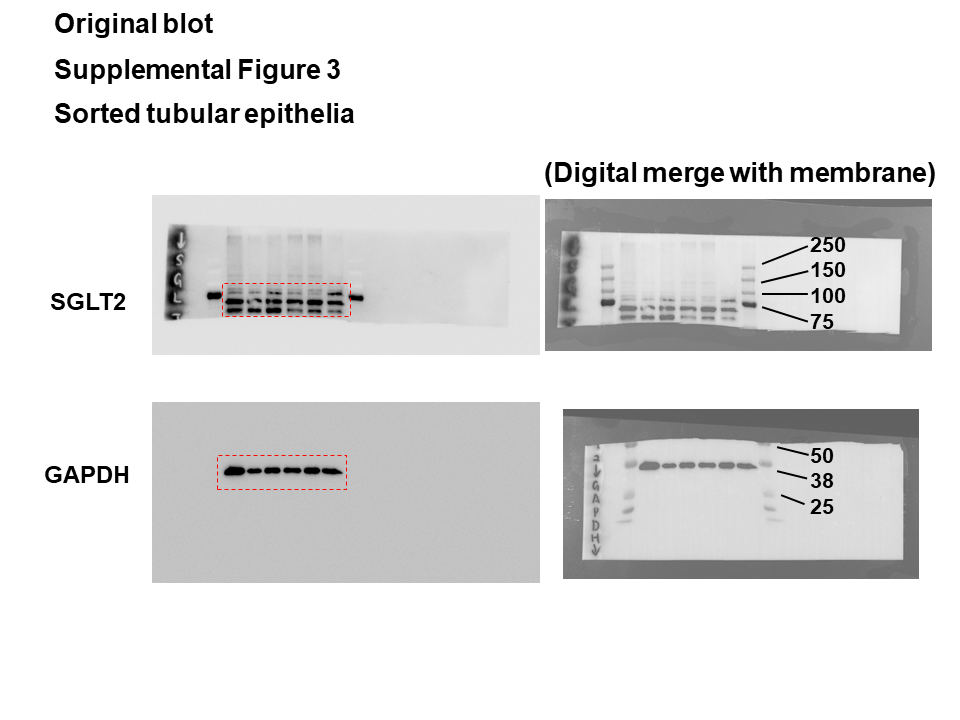
**

**Supplemental Table 1**. The primary and secondary antibodies for immunostaining and western blot

| Immunostaining | Source | Catalog # | Vendor |
| --- | --- | --- | --- |
| **Primary antibody** |  |  |  |
| SGLT2 | rabbit | ab85626 | abcam |
| FITC-conjugated LTL |  | FL1321 | Vector Labs |
| **Dye-conjugated**  **secondary antibody** |  |  |  |
| anti-rabbit antibody | Goat | ab150077 | abcam |

| Western blot | Source | Catalog # | Vendor |
| --- | --- | --- | --- |
| **Primary antibody** |  |  |  |
| SGLT2 | rabbit | ab85626 | abcam |
| GAPDH (HRP-Conjugated) | mouse | ab105428 | abcam |
| **secondary antibody** |  |  |  |
| anti-rabbit antibody | goat | 7074S | CST |

**Supplemental Table 2**. The primers for qPCR

| Gene | Forward | Reverse |
| --- | --- | --- |
| *Slc5a2* | ATGGAGCAACACGTAGAGGC | ATGACCAGCAGGAAATAGGCA |
| *Slc5a1* | ATGCGGCTGACATCTCAGTC | ACCAAGGCGTTCCATTCAAAG |
| *Slc9a3* | CTGAGGAGGAACCGAGCA | AGGCCCAGAACGATGAGTAG |
| *Slc34a1* | ACAAAACCCTACTGGGTGGA | CTCGCTGTAGGACATCAT |
| *Slc2a2* | TCAGAAGACAAGATCACCGGA | GTCATAGCCGAACTGGAAGGA |
| *Slc2a1* | GCAGTTCGGCTATAACACTGG | GCGGTGGTTCCATGTTTGATTG |
| *Vim* | GCTGCGAGAGAAATTGCAGGA | CCATTTCCGTTCAAGGTCAAG |
| *G6pc* | TTACCAAGACTCCCAGGACTG | GAGCTGTTGCTGTAGTAGTCG |
| *Pck2* | CCCCTTGTCTATGAAGCCCTCA | GCCCTTGTGTTCTGCAGCAG |
| *Lrp2* | AAAATGGAAACGGGGTGACTT | GGCTGCATACATTGGGTTTTCA |
| *Havcr1* | AAACCAGAGATTCCCACACG | GTCGTGGGTCTTCCTGTAGC |
| *Pcna* | TTGCACGTATATGCCGAGACC | GGTGAACAGGCTCATTCATCTCT |
| *Fen-1* | ACCAGTTCCTGATTGCTGTTC | TCATGCGGATGGTACGGTAGA |
| *Cdk1* | AGGTACTTACGGTGTGGTGTAT | CTCGCTTTCAAGTCTGATCTTCT |
| *Top2a* | TGCTCCGCCCAGATACCTAC | TGGGTCCCTTTGTTTGTTATCAG |
| *Gapdh* | TGCGACTTCAACAGCAACTC | CTTGCTCAGTGTCCTTGCTG |
| *Actb* | AGCCATGTACGTAGCCATCC | CTCTCAGCTGTGGTGGTGAA |
